# STAT3 is Overactivated in Gastric Cancer Stem-Like Cells 

**DOI:** 10.22074/cellj.2016.3834

**Published:** 2016-01-17

**Authors:** Monireh Hajimoradi, Zuhair Mohammad Hassan, Marzieh Ebrahimi, Masoud Soleimani, Mahdieh Bakhshi, Javad Firouzi, Fazel Sahraneshin Samani

**Affiliations:** 1 Department of Immunology, Faculty of Medical Sciences, Tarbiat Modares University, Tehran, Iran; 2 Department of Stem Cells and Developmental Biology, Cell Sciences Research Center, Royan Institute for Stem Cell Biology and Technology, ACECR, Tehran, Iran; 3 Department of Hematology, Faculty of Medical Sciences, Tarbiat Modares University, Tehran, Iran; 4 Department of Molecular Medicine, Faculty of Advanced Medical Technologies, Gorgan University of Medical Sciences, Gorgan, Iran

**Keywords:** Gastric Cancer, Cancer Stem Cells, Spheroid, STAT3, EMT

## Abstract

**Objective:**

Gastric cancer (GC) is widely associated with chronic inflammation. The pro inflammatory microenvironment provides conditions that disrupt stem/progenitor cell proliferation and differentiation. The signal transducer and activator of transcrip- tion-3 (STAT3) signaling pathway is involved in inflammation and also contributes to the maintenance of embryonic stem cell (ESCs) pluripotency. Here, we have investi- gated the activation status of STAT3 in GC stem-like cells (GCSLCs).

**Materials and Methods:**

In this experimental research, CSLCs derived from the human GC cell line MKN-45 and patient specimens, through spheroid body formation, character- ized and then assayed for the STAT3 transcription factor expression in mRNA and protein level further to its activation.

**Results:**

Spheroid cells showed higher potential for spheroid formation than the pa- rental cells. Furthemore, stemness genes *NANOG, c-MYC* and *SOX-2* were over expressed in spheroids of MKN-45 and in patient samples. In MKN-45 spheroid cells, epithelial mesenchymal transition (EMT) related markers *CDH2, SNAIL2, TWIST* and *VIMENTIN* were upregulated (P<0.05), but we observed no change in expression of the E-cadherin epithelial marker. These cells exhibited more resistance to docetaxel (DTX) when compared with parental cells (P<0.05) according to the MTS assay. Al- though immunostaining and Western blotting showed expression of the STAT3 pro- tein in both spheroids and parents, the mRNA level of STAT3 in spheroids was higher than the parents. Nuclear translocation of STAT3 was accompanied by more intensive phospho-STAT3 (p-STAT3) in spheroid structures relative to the parent cells accord- ing to flow cytometry analysis (P<0.05).

**Conclusion:**

The present findings point to STAT3 over activation in GCSLCs. Com- plementary experiments are required to extend the role of STAT3 in stemness fea- tures and invasion properties of GCSCs and to consider the STAT3 pathway for CSC targeted therapy.

## Introduction

Cancer stem cells (CSCs) are defined as cells capable of giving rise to a new tumor. They are thought to be cancer-initiating cells responsible for cancer relapse and metastasis due to resistance to current therapeutic approaches (1,2). Various efforts to recognize CSCs in many types of cancers provide the opportunity to overcome cancer by targeting specific characteristics of these tumor-initiating cells such as the signaling pathways and their components. Signaling pathways involved in both cancer and stem cells include the Janus kinase (JAK)/signal transducer and activator of transcription-3 (STAT), Hedgehog (Hh), epidermal growth factor (EGF), Notch, mitogen-activated protein kinase (MAP-Kinase)/extracellular signal-regulated kinases (ERK), phosphatidylinositol 3 kinase (PI3K)/acutely transforming retrovirus (AKT), nuclear factor kappa β (NFkβ), Wnt and the transforming growth factor beta (TGF-β) signaling pathways (3,4). However, revealing the signaling networks required for the maintenance of stemness in CSCs can be exploited in cancer treatment. It has been reported that some of these pathways are highly activated in CSCs relative to the bulk of tumors in a wide variety of human cancers. 

STAT3, initially identified as a mediator of the inflammation-associated acute phase response, is considered an oncogene (5). Physiologically, STAT3 is activated only transiently. In many cancers, by contrast, STAT3 is constitutively activated in a portion of the tumor bulk, (6) including cancers of the breast, prostate, ovary, pancreas and leukemia (7,11), as well as gastric cancer (GC) (12). The activation state is frequently related to a poor prognosis (13). On the other hand, STAT3 in cooperation with NANOG maintains embryonic stem cell pluripotency (14,15) and is essential for preservation of stemness characteristics, proliferation and tumorigenicity of CSCs in breast cancer and glioblastoma (16,17). 

Growing evidences suggest that inflammation promotes epithelial mesenchymal transition (EMT) (18). Numerous observations have also described the role of STAT3 in regulation of cellular proliferation, invasion, migration, and angiogenesis (19). Metastasis accounts for over 90% of cancer mortality (20) and is dependent on EMT and its counterpart process, mesenchymal epithelial transition (MET). CSCs have shown a correlation with the EMT process (21). 

In GC, some putative cell surface marker have been introduced to identify CSC subpopulations. Among the most consistent markers are CD44 (22,25), EpCAM (23), CD24 (24), and CD133 (25,26). Side population (SP) cells and spheroid structures from GC cell lines and primary GC tissues have also been proposed as cancer stem-like cells (CSLCs) (23,27,28). 

The main purpose of this study was to investigate the level of STAT3 activation subsequent to isolation of CSLCs from GC, which was representative of chronic inflammationinduced cancers. Therefore, GCSLCs were enriched and characterized human GC cell line MKN-45 and patient tumor specimens based on sphere culture. Next we assessed STAT3 activity by examining phospho-STAT3 (p-STAT3), the active form of STAT3. Further confirmation of the present findings might suggest that the STAT3 pathway could be a component of the stemness signaling network in CSCs. 

## Materials and Methods

The patient gastric adenocarcinoma samples were provided by the Iranian National Tumor Bank, the Cancer Institute of Iran, Imam Hospitals Complex. Informed consents were obtained from participant patients and the Royan Institute’s Institutional Review Board approved the project. 

## Cultures of cells and tumor tissues

For this experimental research, tumor samples were obtained within 1-2 hours after surgical resection from two adult GC patients (GC 19 and GC 24). Tumor tissues were washed and mechanically dissociated into small (1-2 mm^3^) fragments. Tissue fragments of tumor specimens and the human gastric adenocarcinoma cell line MKN-45 were cultured in Roswell Park Memorial Institute medium (RPMI)-1640 supplemented with 10% fetal bovine serum (FBS, Gibco, USA), 100 U/ml penicillin and 100 μg/ml streptomycin (both from Gibco, USA) as an adherent monolayer culture and were trypsinized to a single cell preparation and further passages. For tissue specimens, 50 μg/ml of gentamicin (Sigma, USA) and 0.25 ng/ml of amphotericin-B (Gibco, USA) were added to the culture medium. The passage number of cultured cells was not more than 5.

## Gastrosphere formation and spheroid formation efficacy 

In order to generate a primary culture of floating spheroids with enriched cancer stem cells, we placed MKN-45 cells at a density of 10000 cells/ml in serum-free RPMI that contained B27 (1:50, Gibco, USA), 20 ng/ml of basic fibroblast growth factor (bFGF, Royan Institute, Iran), and epidermal growth factor (EGF, Royan Institute, Iran) in T-25 non-adhesive poly(2-hydroxyethyl methacrylate) (poly-HEMA, Sigma, USA) coated flasks. Spheroids were passaged approximately every week. The spheroids were dissociated enzymatically with accutase (Gibco, USA) into single cells and their capacity to generate secondary and tertiary gastrospheroids was examined by counting the spheroids larger than 50 μm at the time of passage in 10 visualized fields under a microscope. Fragments of tumor specimen were cultured in the same conditions as MKN-45 to form spheroids. To compare spheroid formation efficacy of adherent culture and spheroids derived from patient samples, single cells were seeded at 2000 cells/ well in six-well ultra-low attachment plates (Corning, USA). After approximately 7 days, the spheroids were counted. 

## Chemoresistancy by MTS colorimetric cell proliferation assay

In order to prove the increased chemoresistance of spheroids compared to MKN-45 parental cells, we used the MTS assay as described below. Single cells of spheroids and parental cells were treated for 72 hours with docetaxel (DTX, Sigma, USA) at 0.5 μM concentration, which we determined to be the 50% inhibitory concentration (IC50) of DTX on MKN-45 cells. In order to determine the estimated IC50, we seeded single cells (5000 cells/well) of the MKN-45 monolayer culture onto 96-well plates. Cells were treated with different doses (0.5-100 μM) of DTX in triplicate. Dimethyl sulfoxide (DMSO, Sigma, USA), a DTX solvent, was considered the negative control. At 24, 48 and 72 hours post-treatment, 10 μl of MTS solution (Promega, USA) was added to the wells and incubated for 3 hours. Next, we measured the absorbance at 490 nm in order to determine cell viability. 

## Phospho flow cytometry

Phospho flow cytometry analysis of p-STAT3 was performed in order to examine whether the amount of activated STAT3 was increased in spheroid cells. Dissociated single cell suspensions were prepared enzymatically from adherent and spheroid cultures of MKN-45 and tumor tissue samples, then washed and fixed by the direct addition of phosphate buffered saline (PBS)/paraformaldehyde (PFA) into the culture medium in order to obtain a final concentration of 1.5% PBS/PFA for 10 minutes at room temperature and pelleted. The cells were subsequently permeabilized by suspending with vigorous vortexing in 500 µl ice cold methanol and incubated in ice for 30 minutes. After three times washing in PBS/ bovine serum albumin (BSA) (0.5%), the cells were stained with the primary antibody (monoclonal rabbit anti-pTyr 705-STAT3, 1:100, Cell Signaling) for 45 minutes at room temperature, then washed and incubated with Alexa Fluor-488 labeled goat antirabbit IgG (1:500, Invitrogen, USA) for 30 minutes at 37˚C. Cells stained only with the secondary antibody were considered to be the negative control. Flow cytometry analysis of the cell populations were performed with a BD FACS-Calibur flow cytometer and Flowing software was used for data analyses. 

## RNA extraction and quantitative real-time polymerase chain reaction

Cells were collected and preserved at -80˚C until RNA extraction. Total RNA was isolated using Trizol reagent (Qiagen, USA) and treated with DNAse I (Fermentas, USA) for 30 minutes in order to digest the genomic DNA. The quality of RNA samples was monitored by agarose gel electrophoresis and a spectrophotometer (Biowave ІІ, UK). A total of 2 μg of RNA was reverse transcribed with a cDNA synthesis kit (Fermentas, USA) and random hexamer primers according to the manufacturer’s instructions. Transcript levels were determined using the SYBR Green master mix (Takara, Japan) and a Rotorgene 6000. Expression of genes involved in stemness features and genes that regulate the process of EMT were normalized to the *GAPDH* housekeeping gene. Relative quantification of gene expression was calculated using the ∆∆Ct method. Primer sequences for quantitative real-time polymerase chain reaction (qRT–PCR) are listed in table 1. 

**Table 1:** Primer sequences used for quantitative real-time polymerase chain reaction


Primername	Sequence

*OCT4*	F:5´GTTCTTCATTCACTAAGGAAGG3´
	R:5´CAAGAGCATCATTGAACTTCAC3´
*SOX2*	F:5´GGGAAATGGAAGGGGTGCAAAAGAGG3´
	R:5´TTGCGTGAGTGTGGATGGGATTGGTG3´
*KLF4*	F:5´ACGATCGTGGCCCCGGAAAAGGACC3´
	R:5´TGATTGTAGTGCTTTCTGGCTGGGCTCC3´
*c-MYC*	F:5´GCGTCCTGGGAAGGGAGATCCGGAGC3´
	R:5´TTGAGGGGCATCGTCGCGGGAGGCTG3´
*NANOG*	F:5´CAGCTACAAACAGGTGAAGAC3´
	R:5´TGGTGGTAGGAAGAGTAAAGG3´
*STAT3*	F:5´GAAGAATCCAACAACGGCAG3´
	R:5´TCACAATCAGGGAAGCATCAC3´
*GAPDH*	F:5´GAAATCCCATCACCATCTTCC3´
	R:5´GGCTGTTGTCATACTTCTCAT3´
*CDH1*	F:5´GCTCTCCACTCTTACTTCCT3´
	R:5´GTTTGGTCTGATGCG3´
*CDH2*	F:5´GCCCAAGACAAAGAGACCC3´
	R:5´CTGCTGACTCCTTCACTGAC3´
*TWIST1*	F:5´CCAGGTACATCGACTTCCTC3´
	R:5´TCGTGAGCCACATAGCTG3´
*SNAIL1*	F:5´CCAGAGTTTACCTTCCAGCA3´
	R:5´GATGAGCATTGGCAGCGA3´
*SNAIL2*	F:5´AACTACAGCGAACTGGACAC3´
	R:5´GGATCTCTGGTTGTGGTATGAC3´
*VIMENTIN*	F:5´AAACTTAGGGGCGCTCTTGT3´
	R:5´TGAGGGCTCCTAGCGGTTTA3´


## Immunofluorescent staining of STAT3

Immunocytofluorescent staining was performed to assess the expression of STAT3 at the protein level and its localization. Briefly, MKN-45 cells cultured in 96-well plates were fixed with 4% PFA for 20 minutes at room temperature, washed with PBS/5% Tween, and permeabilized with 0.5 % triton-X100. After washing, the cells were blocked in 10% goat serum/PBS, and stained with polyclonal rabbit anti-STAT3 (1:100, Santa Cruz). Cells were stained with a secondary Alexa Fluor-488 labeled goat anti-rabbit IgG (1:500, Sigma, USA) and counterstained with 1 μg/ml 4,6-diamino2-phenyl indole dihydrochloride (DAPI, Sigma, USA). Cells were visualized using an Olympus fluorescent microscope. In the case of MKN-45 spheroids, paraffin-blocks were prepared from agarose embedded formalin-fixed spheroids after which 5 μm sections from the blocks were examined by immunostaining. Paraffin sections were subjected to antigen retrieval for 30 minutes at 95˚C, deparaffinized in xylene, and rehydrated in a series of graded methanol. The slides were subsequently stained as described for adherent MKN-45 cells for STAT3. Additional slides were stained in a standard manner with hematoxylin and eosin (H&E). 

## Western blot analysis

Protein extracts were obtained from 10^6^cells by lysis in Trizol (Qiagen, USA) that contained protease inhibitors (Sigma, USA). Cell lysates (20 μg) were separated on 10% sodium dodecyl sulfate (SDS)-polyacrylamide gel and then transferred to Polyvinylidene fluoride (PVDF) membranes (Bio-Rad, USA). The blots were blocked with 5% BSA in Tris buffered saline with tween (TBST, 20 mM Tris–HCl, pH=7.6, 150 mM NaCl, and 0.1% Tween-20), and then incubated overnight at 4˚C with polyclonal rabbit anti-STAT3 primary antibody (1:2000, Santa Cruz, USA) and 1 hour at room temperature for GAPDH (Sigma, USA). After washing with TBST, the membranes were incubated with anti-rabbit horseradish peroxidase (HRP)-conjugated secondary antibody (Sigma, USA) for 45 minutes at room temperature. Protein bands were visualized with ECL substrate (GE) on Hyperfilm (GE). *GAPDH* was used as the control for normalization. 

## Statistical analysis

Data were expressed as mean ± SD/SEM of at least three independent replicates. Statistical comparisons between two groups were made using one-way ANOVA, the student’s t test or nonparametric Mann-Whitney U test. P<0.05 was considered statistically significant. 

## Results

### Gastrospheroids characterized as gastric cancer stem-like cells

**Fig.1:**
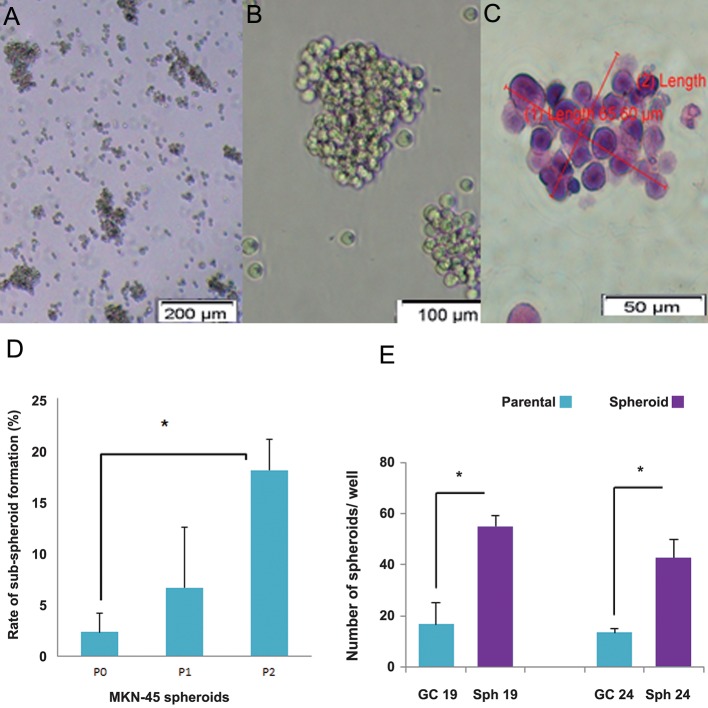
Enrichment of cancer stem-like cells based on serial spheroid formation. A. Single cells from patient specimen, B. MKN-45 cell line cultivated in SFM supplemented with EGF, bFGF and B27 and non-adhesive conditions formed spheroid structures, C. H&E staining of MKN-45 spheroids, D. In MKN-45, sub-culture of spheroid cells resulted in increased potential for spheroid formation and cancer stemlike cell enrichment with increased numbers of passages and E. Numbers of spheroids from 2000 cells seeded per well of a six-well plate which shows the increased potential for spheroid formation in spheroids passage 3 compared to the parental cells. Data are mean ± SD. *; P<0.05, SFM; Serum-free medium, EGF; Epidermal growth factor, bFGF; Basic fibroblast growth factor, GC; Gastric cancer and H&E; Hematoxylin and eosin.

The capability to form spheroid structures, a characteristic of embryonic stem cells, was used to enrich the cells with stemness properties within the cancer cells. MKN-45 single cells and tumor tissue fragments in defined serum free medium (SFM) supplemented with EGF, bFGF and B27 formed bodies that resembled spheres which were loosely attached cells that had a grape-like shape (Fig .1A-C). The spheroids were continuously passaged to form subspheroids. Spheres at passages 3 to 5 were used for further analyses. As shown in figure 1D, the rate of spheroid formation increased with increasing passage number (P<0.05). In the MKN-45 cell line this rate was 2.30% in MKN45 parental cells and increased to 18.03% in passage-2 MKN-45 spheroids. Also, passage-3 spheroids derived from GC 19 and GC 24 patient specimens were more potent in spheroid formation than the monolayer culture (Fig .1E). 

**Fig.2:**
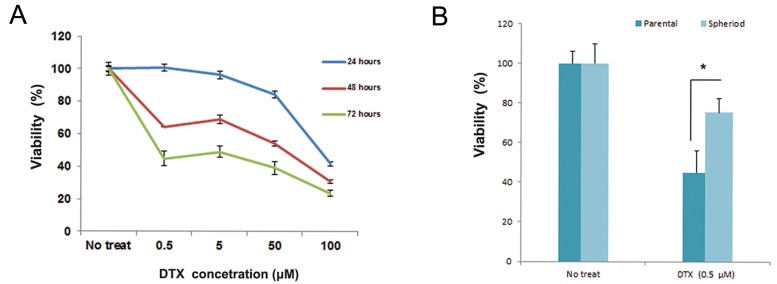
Drug resistancy of MKN-45 spheroids to DTX by the MTS assay. A. Dose-response curve to determine the IC50 of DTX in the MKN- 45 cell line at 24, 48, and 72 hours and B. MKN-45 spheroids showed increased resistance to 0.5 μM DTX after 72 hours of treatment compared to parental cells. Data are mean ± SD. *; P<0.05, DTX; Docetaxel and IC50; Inhibitory concentration 50.

We tested the drug resistance potential of GCSLCs and the parental cells by adding 0.5 μM of DTX (Fig .2A,B) to the single cells derived from spheroids or the monolayer culture of MKN-45 for 72 hours. Spheroid cells were significantly resistant to the cytotoxic effect of DTX (P<0.05) compared to MKN-45 parental cells. 

**Fig.3:**
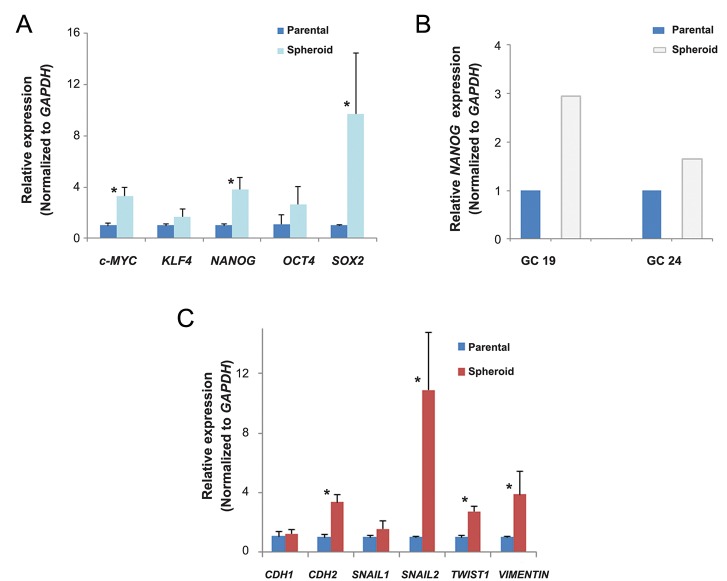
Examination of stem cell markers and EMT-related mRNA expression by qRT-PCR. A. Spheroids derived from MKN-45, B. Patient specimens over expressed a number of stemness transcription factor genes compared to parental cells and C. mRNA levels of *CDH2, SNAIL2, TWIST* and *VIMENTIN* genes contributed to the EMT process were up regulated in MKN-45 spheroids compared to parental cells. Data are mean ± SEM. *; P<0.05, EMT; Epithelial mesenchymal transition, GC; Gastric cancer and qRT-PCR; Quantitative real-time polymerase chain reaction.

We have evaluated whether spheroids overexpressed stems cell markers and EMT related genes that represented higher invasive capacity by qRTPCR. *NANOG, OCT4, SOX2, c-MYC* and *KLF4* are engaged in the regulation of embryonic stem cell pluripotency and their involvement in maintenance of stemness features of different types of CSCs has recently been discovered. Our results determined that *NANOG, c-MYC* and *SOX2* significantly up regulated (P<0.05) in spheroid derived cells from MKN-45 compared to parental cells. Spheroids of GC 19 and GC 24 patient samples also had increased *NANOG* expression (Fig .3A,B). The expressions of EMT regulatory markers *CDH2, SNAIL2, TWIST* and *VIMENTIN* were increased in MKN-45 gastrospheres (Fig .3C,P<0.05). However, the expression of *CDH1*, an epithelial marker, did not change in spheroid derived cells. 

### Gastric cancer stem-like cells had an enhanced level of STAT3 activation

**Fig.4:**
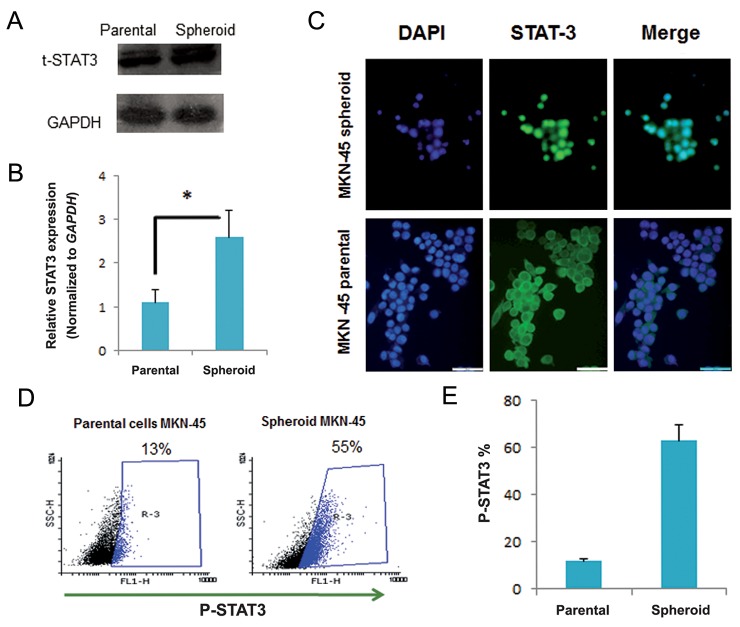
STAT3 expression and activation in gastric cancer stem-like cells. A. Spheroid cells displayed higher expression of STAT3 at the protein, B. mRNA levels compared to parental cells in MKN-45 cells, C. Immunostaining of STAT3 in MKN-45 derived spheroids showed nuclear translocation which is related to phosphorylation and activation, D. Flow cytometric analysis of p-STAT3 in MKN-45 cells and the derivative spheroids in which STAT3 is stained with anti p-STAT3 antibody and Ax-488 labeled secondary antibody and E. STAT3 is significantly more phosphorylated in spheroids compared to MKN-45 parental cells. Data are mean ± SEM. *; P<0.05, t-STAT3; Total-STAT3 and p-STAT3; Phospho-STAT3.

**Fig 5:**
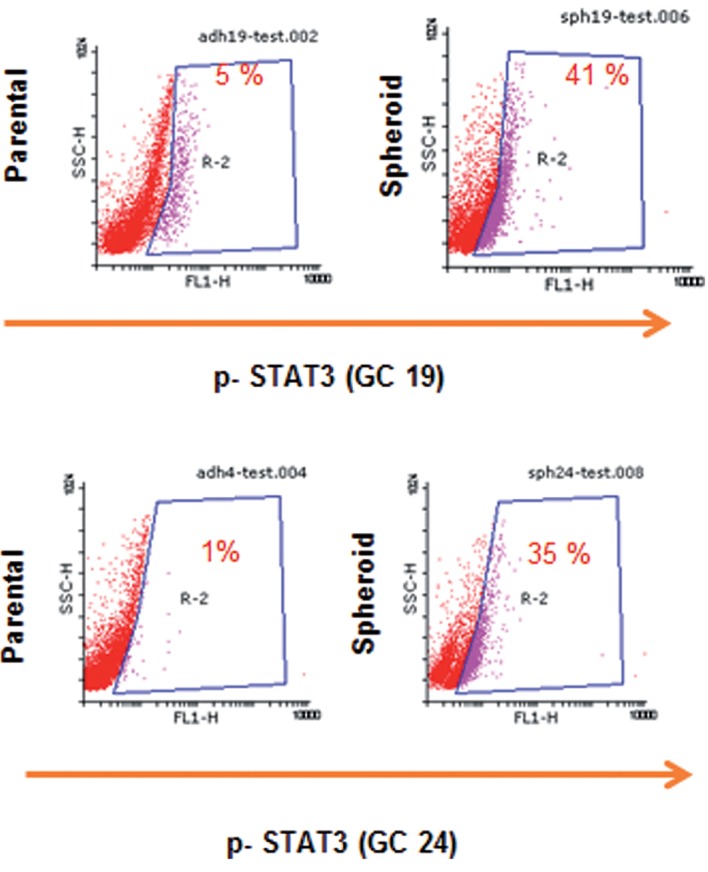
Flow cytometric analysis of phospho-STAT3 (p-STAT3) in tumor tissue samples. Representative plots of p-STAT3 staining with primary anti-p (Tyr 705) -STAT3 and Ax-488 labeled secondary antibody. P-STAT3 is more frequent in GC 24 (41%) and GC 19 (35%) spheroids compared to GC 19 (5%) and GC 24 (1%) parental cells. Tyr 705; Tyrosine 705 and Ax-488; Alexa fluor-488.

In order to determine the presence of STAT3 activation, as an important signaling pathway in CSC self-renewal, we performed qRT–PCR, Western blot, immunostaining and flow cytometric analyses. As shown in figure 4A, STAT3 at the protein level expressed in both spheroids and MKN-45 monolayers. However, its mRNA level was more than two-fold higher in MKN-45 spheroids compared to parental cells (Fig .4B). Localization of STAT3 is dependent on its activation status. STAT3 localize in the cytoplasm, whereas its active form, p-STAT3, translocates to the nucleus. Immunostaining for STAT3 indicated that STAT3 was localized within the nucleus in spheroids. However in MKN-45 monolayers, STAT3 had a more intense cytoplasmic location compared to the nucleus (Fig .4C). Phosphoflowcytometry of p-STAT3 was performed to evaluate the activation status of the STAT3 transcription factor. The spheroid group of MKN-45 cell was 63% positive for p-STAT3 which was significantly (P<0.05) higher than the parental control group, which was 12% (Fig .4D,E). Analysis of spheroids derived from specimens GC 19 and GC 24 showed that 42% of GC 19 and 35% of GC 24 were p-STAT3 positive, while only 5 and 1% were respectively located in the monolayer culture of the related tissue (Fig .5). 

## Discussion

The link between infection, chronic inflammation, and cancer has long been recognized (29). Approximately 25% of cancers emerge due to chronic infection or other types of chronic inflammation (30). *Helicobacter pylori (H.pylori)* induced inflammation, as an example, is a major cause of GC (31). It is currently accepted that all solid tumors contain an inflammatory microenvironment (32). *H.pylori*, classified as a class I carcinogen (33), induces local chronic inflammation and persistent activation of multiple oncogenic signaling within the gastric epithelium (34). The proinflammatory microenvironment may deregulate stem/progenitor cell proliferation and differentiation (4), finally resulting in transformation of stem or progenitor cells to cancer stem cells (35). However, a number of reports have stated that GC may originate from bone marrow derived stem cells (BMDSCs) that migrate to inflammatory sites in the stomach (36). In attention to rigorous relationship between GC and inflammation and the role of STAT3 in inflammation, we have aimed to determine the status of STAT3 in the initiation of GC by conducting an evaluation of its activation in GCSLCs. 

Contradictory reports on specification of CSCs and no consensus to define the best cell surface markers for GCSCs have encouraged us to enrich these cells according to a more definite functional assay-spheroid formation. Accumulating evidence suggests that spheroid structures derived from various cancers and cell lines known to be enriched with CSLCs as in GC (23,37,38). Our data supported the results from other reports which strongly suggested that spheroid structures could be indicators of putative cancer stem cells in the human GC cell line MKN-45 and GC tissue samples. The characteristics of spheroid cells were investigated with respect to spheroid-forming capacity, chemoresistancy to DTX, expression of pluripotency, and EMT markers. Compared with parental cells, the spheroid derived cells were more chemoresistant. DTX is administered to advanced GC patients either as a single agent or in combination with other chemotherapeutic agents. However, the response rates rarely exceed 40-45% (39). This decreased responsiveness or disease progression may be due to the presence of cells that have stemness features. 

Contradictory reports on specification of CSCs and no consensus to define the best cell surface markers for GCSCs have encouraged us to enrich these cells according to a more definite functional assay-spheroid formation. Accumulating evidence suggests that spheroid structures derived from various cancers and cell lines known to be enriched with CSLCs as in GC (23, 37, 38). Our data supported the results from other reports which strongly suggested that spheroid structures could be indicators of putative cancer stem cells in the human GC cell line MKN-45 and GC tissue samples. The characteristics of spheroid cells were investigated with respect to spheroid-forming capacity, chemoresistancy to DTX, expression of pluripotency, and EMT markers. Compared with parental cells, the spheroid derived cells were more chemoresistant. DTX is administered to advanced GC patients either as a single agent or in combination with other chemotherapeutic agents. However, the response rates rarely exceed 40-45% (39). This decreased responsiveness or disease progression may be due to the presence of cells that have stemness features. Chemotherapy accompanied by approaches that target these cells would be promising approaches. In a study by Liu et al. (37), gastrospheres of MKN- 45 also exhibited resistance to 5-fluorouracil (5- FU) and DDP (cisplatin). They also overexpressed *OCT-4, SOX2, NANOG* and *CD44*.

We have found that GCSLCs had more similar characteristics to embryonic stem cells (ESCs) in the elevated expressions of some pluripotency factors *SOX-2, NANOG* and *c-MYC*. It has been previously shown that the JAK/STAT3 pathway is important in the pluripotent state of murine ESCs (mESCs), mainly through activation of *c-MYC* and *KLF4* (40). SOX2 in concert with OCT4 binds to promoters *STAT3* and *NANOG* (41). According to the present findings, the elevated level of SOX2 in GCSCs could be one of the underlying mechanisms for *STAT3* gene overexpression. Previous study has also shown that NANOG functions in parallel to STAT3 in the maintenance of stem cell properties (42). Bourguignon et al. (43) uncovered a functional link between *NANOG* and *STAT3*. These researchers reported that hyaluronan (HA) binding to CD44 induced *NANOG* activation and its interaction with STAT3 which resulted in STAT3 specific transcriptional activation, MDR1 gene expression, and tumor cell growth in human breast and ovarian tumor cells. They reported that HA/CD44 signaling through *NANOG/STAT3* promoted *MIR-21* expression and further resulted in anti-apoptosis as well as chemoresistance in head and neck squamous cell carcinoma (HNSCC) cells (40). Of note, the miR-21 promoter contains STAT3 binding site(s). A number of miRs are differentially expressed in GCSCs and cancer cells. These include miR-21 and let-7a (44). STAT3 controls expression of some miRs and identified putative STAT3 binding sites in promoter regions of miRs, including miR-21 (45, 46). Hence, evaluation of the *CD44/NANOG/STAT3* axis in regulation of specific miRs that control self-renewal in GCSCs seems to be valuable.

We illustrated here, the higher activation of STAT3 in GCSLCs by conducting an examination of p-STAT3 and its nuclear location. Concomitant with STAT3 overactivation, as a proinflammatory transcription factor, we have observed upregulation of some EMT markers (*CDH2, SNAIL2, TWIST* and *VIMENTIN*) which are representative of the mesenchymal phenotype and associated with invasion. There was no change in E-cadherin expression level, which indirectly depends on STAT3 (47, 48). STAT3 binds to and significantly activates the *TWIST* promoter in cooperation with EGF receptor (EGFR) in human breast cancer cells (49).

In agreement with the findings by Yang et al. (50) our results provided evidence that GCSCs might be involved in GC invasion and metastasis through EMT. A crucial role for STAT3 in the EMT process comes from the fact that EMT-related transcription factors such as ZEB, TWIST and SNAIL are activated by STAT3. Subsequently these transcription factors down-regulate E-cadherin expression (47, 48). Other mechanisms proposed for STAT3 mediated regulation of invasion include control of WASF3 protein activation (51) or through induction or suppression of effector miRs expression. MiR-34a is directly repressed by STAT3 and it has been demonstrated that in colorectal cancer an active IL-6R/STAT3/miR-34a loop is necessary for EMT, invasion and metastasis (52).

Thus, our recent experiments in accordance with previous evidences have shown the potential roles of the STAT3 signaling pathway in development of chemoresistance or regulation of EMT in GCSCs. Based on the investigation on glioblastoma and breast cancers, STAT3 overactivation has been recognized to be essential for proliferation, sphere formation, EMT and tumorigenesis of CSCs (16, 17, 53, 54).

## Conclusion

Spheroid formation provides an applicable method to isolate GCSLCs from the MKN-45 cell line and probably from tissue samples. We have found that gastrospheroids resembeled as GCSLCs, express STAT3 that is phosphorylated on the activating tyrosine (Tyr 705) and has an intranuclear localization. It is necessary to discover the definitive markers that identify GCSCs. Further confirmation that aberrant activation of STAT3 is effective in stemness and invasion of GCSLCs through STAT3 inhibition and the definite mechanism of action of GCSCs through examination of more tissue samples and additional *in vivo* experiments.
